# Identification of a Prognostic Signature Associated With DNA Repair Genes in Ovarian Cancer

**DOI:** 10.3389/fgene.2019.00839

**Published:** 2019-09-12

**Authors:** Hengzi Sun, Dongyan Cao, Xiangwen Ma, Jiaxin Yang, Peng Peng, Mei Yu, Huimei Zhou, Ying Zhang, Lei Li, Xiao Huo, Keng Shen

**Affiliations:** Department of Obstetrics and Gynecology, Peking Union Medical College Hospital, Chinese Academy of Medical Sciences and Peking Union Medical College, Beijing, China

**Keywords:** ovarian cancer, DNA damage response, genome integrity, prognostic signature, therapeutic target

## Abstract

**Introduction:** Ovarian cancer is a highly malignant cancer with a poor prognosis. At present, there is no accurate strategy for predicting the prognosis of ovarian cancer. A prognosis prediction signature associated with DNA repair genes in ovarian cancer was explored in this study.

**Methods:** Gene expression profiles of ovarian cancer were downloaded from the GEO, UCSC, and TCGA databases. Cluster analysis, univariate analysis, and stepwise regression were used to identify DNA repair genes as potential targets and a prognostic signature for ovarian cancer survival prediction. The top genes were evaluated by immunohistochemical staining of ovarian cancer tissues, and external data were used to assess the signature.

**Results:** A total of 28 DNA repair genes were identified as being significantly associated with overall survival (OS) among patients with ovarian cancer. The results showed that high expression of XPC and RECQL and low expression of DMC1 were associated with poor prognosis in ovarian cancer patients. The prognostic signature combining 14 DNA repair genes was able to separate ovarian cancer samples associated with different OS times and showed robust performance for predicting survival (Training set: p < 0.0001, AUC = 0.759; Testing set: p < 0.0001, AUC = 0.76).

**Conclusion:** Our study identified 28 DNA repair genes related to the prognosis of ovarian cancer. Using some of these potential biomarkers, we constructed a prognostic signature to effectively stratify ovarian cancer patients with different OS rates, which may also serve as a potential therapeutic target in ovarian cancer.

## Introduction

Ovarian cancer is the fifth leading cause of cancer-related death in women (Siegel et al., 2018). The average lifetime risk of developing ovarian cancer is 1.3%, and the 5-year survival rate ranges from 29% to 93% depending on the spread of the cancer at diagnosis (Torre et al., 2018). In developed countries, the mortality rate of ovarian cancer has declined only slightly over the past 3 decades, despite advances in treatment strategies and techniques, largely because nearly 60% of cases are diagnosed at an advanced stage due to a lack of obvious symptoms and adequate screening tests (Siegel et al., 2018). Standard treatment for advanced disease involves debulking surgery and chemotherapy. However, most patients experience relapse within 2 or 3 years after receiving the first-line chemotherapy regimen and die due to chemoresistance (Dinh et al., 2008). Considering the critical role that tumor molecular biology plays in the initiation and progression of tumors, researchers and clinicians have to date focused on effective targeted prognostic and treatment strategies for ovarian cancer (Klinck et al., 2008).

Genomic instability is a hallmark of cancer. Cells develop a complex DNA damage response (DDR) to repair DNA damage and promote maintenance of genome integrity. Defects in DDR are associated with failure to accurately repair damaged DNA in cells, leading to the transformation of normal cells into cancer cells with accumulated genetic changes (Minchom et al., 2018). Due to defects in DDR, cancer cells often show a reduced capacity to repair DNA compared to normal cells and are more reliant on other subsets of repair pathways for survival, which results in DNA replication stress and accumulation of DNA damage. Indeed, approximately 50% of epithelial ovarian cancers are characterized by inactivation of genes required for homologous recombination (Spriggs and Longo, 2018). BRCA 1/2 is well known for its role in DNA repair of double-stranded breaks and recombination (Spriggs and Longo, 2018). Moreover, BRCA mutations have been demonstrated to be associated with improved overall survival (OS) and progression-free survival (PFS) in patients with ovarian cancer. Although the reason remains unclear, it may be due to “synthetic lethality”, which leads to cell death due to a synergistic effect in an already DDR-deficient background, such as PARP inhibition (Kaelin, 2005; Moeller et al., 2009). Synthetic lethality between repair pathways also has provided advanced clinical strategies for targeting DNA repair/DDR (Brown et al., 2017). Such issues render DDR targeting a powerful prognostic strategy.

Therefore, we integrated expression profiling based on the Gene Expression Omnibus (GEO), UCSC Xena, and The Cancer Genome Atlas (TCGA) databases to explore DNA repair genes related to the prognosis of ovarian cancer and potentially to explore DDR targeting biomarkers to improve the survival of ovarian cancer patients.

## Materials and Methods

### Data Sources

After obtaining expression data from the GEO database (https://www.ncbi.nlm.nih.gov/geo/), we integrated 2 sets of data, GSE14001 and GSE14407, containing raw data for 32 ovarian cancer samples and 15 para-carcinoma samples. The HT_HG-U133A dataset from UCSC Xena (https://genome-cancer.ucsc.edu/), which contains 565 samples with prognostic follow-up information, was used to analyse the relationship between the DNA repair gene expression profile and prognosis. Finally, we downloaded another set of RNA-Seq data from TCGA (the July version) containing 379 samples and 364 samples with available follow-up information as an independent validation dataset for survival analysis.

### Data Pre-Processing and Differential Expression Analysis

The data were preprocessed as follows. 1) The original chip data of the GEO dataset were downloaded. 2) The R package (R 3.4.0 version) affy was used to process the chip raw data. 3) RMA standardization was performed to convert the data into expression spectrum chip data. 4) The sva combat method of the R package was further employed to remove the batch effect, and probes were then mapped to the genes. The no-load probe was removed, and multiple probes were designed to correspond to a median of 1 gene. 5) Each dataset was further quantified by quantile normalization to extract expression profiles for the DNA repair genes. 6) We screened DNA repair genes in samples with different grade levels by the Wilcoxon rank test and analyzed genes with expression levels that were significantly different among samples with low, high and normal levels using ANOVA, with p-values <0.05 indicating significant differences. 7) Hierarchical clustering was performed for cluster analysis of the DNA repair gene expression profile for each sample, and the prognoses for samples with different expression patterns were examined. 8) Univariate analysis was performed using the R package survival, and a gene with a significance level of p < 0.05 was selected as a prognostic DNA repair gene. 9) Ovarian cancer molecular subtypes were constructed by unsupervised clustering based on prognostic DNA repair genes, and multivariate Cox proportional hazards models were applied to observe their impacts on prognosis. Next, stepwise regression was applied to establish a prognostic signature. 10) Validation was performed using external TCGA (July version) RNA-Seq data containing 379 samples and 364 samples with follow-up information.

### Immunohistochemical Staining (IHC)

We collected a total of 200 human ovarian tissue samples, 160 of which had accompanying follow-up information, and 40 cancer-adjacent ovarian tissue samples from archives of paraffin-embedded tissues between February 2009 and February 2013 at the Department of Pathology of Peking Union Medical College Hospital. The follow-up was performed until March 30, 2018. The pathological diagnoses were reconfirmed by a pathologist. The project was approved by the Ethical Committee (Peking Union Medical College Hospital), and informed consent was acquired from patients or family members. IHC was performed as previously described (Li et al., 2010). Antibodies against the following were used: XPC 1:200 abcam ab203693; RECQL 1:50 abcam ab203693; DMC1 1:200 abcam ab203693. The scoring details have been described previously (Zhang et al., 2015). The intensity of immunostaining was graded as follows: 1+, weak; 2+, moderate; 3+, strong or 4+, very strong. The area of positive cancer cells in each microscopic field was categorized as follows: 1+, 0 to 25%; 2+, 25 to 50%; 3+, 50 to 75% or 4+, 75 to 100%. The sum between 5 and 80 was obtained by multiplying the 2 scores by 5. A sum from 0 to 42 was assigned as “low expression” and that from 43 to 80 as “high expression.” All pathological diagnoses were confirmed in a blinded manner by 3 expert pathologists.

## Results

### Data Standardization

Two sets of raw chip data (GSE14001 and GSE14407) were downloaded from GEO: GSE14001 contains 10 low-grade ovarian serous carcinoma samples, 10 high-grade ovarian serous carcinoma samples and 3 short-term primary cultures of human ovarian cells; GSE14407 contains 12 ovarian surface epithelial cell lines and 12 ovarian cancer epithelial cell lines. We standardized the raw chip data using the R software package RMA method, and the standardized results are shown in **Supplementary S1a**. The batch effect was further removed using the R package sva combat method, and the results are shown in **Supplementary S1b**. Finally, the results of quantile normalization are shown in **Supplementary Figure S1C**. The testing set that included 727 DNA repair genes was obtained from the DNA repair-related pathways in KEGG and the literature (**Supplementary S2**).

### Expression Patterns of DNA Repair Genes in High-Grade Ovarian Cancer, Low-Grade Ovarian Cancer, and Normal Samples

**Figure 1:**
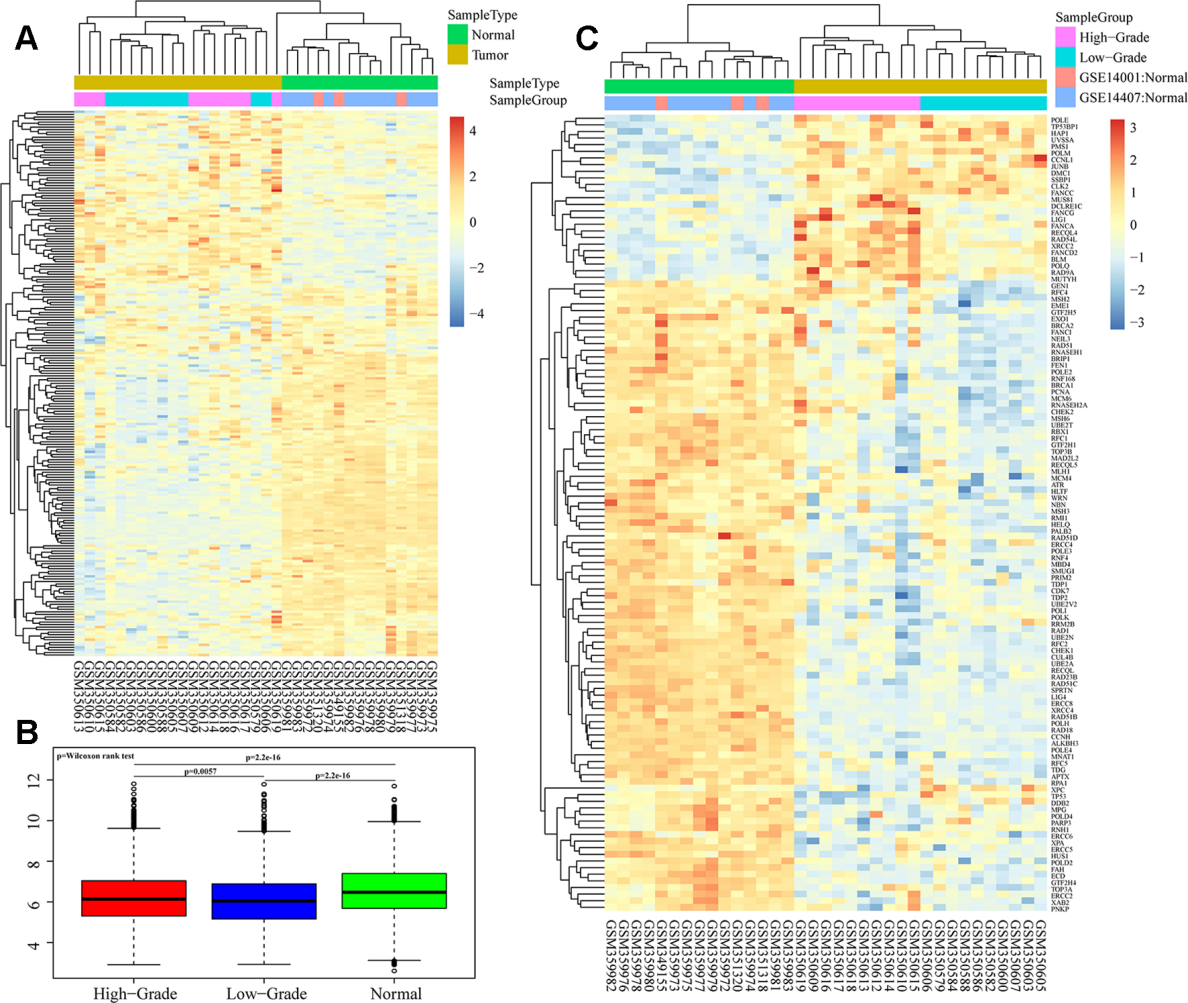
Heatmaps of DNA repair gene expression between ovarian cancer and normal samples in GSE14001 and GSE14407. **(A** and **B)** Overall expression differences of DNA repair genes in high-grade, low-grade and normal groups. **(C)** Cluster analysis of 120 differential gene profiles by ANOVA.

We analyzed overall expression differences in DNA repair genes in the different groups. As shown in **Figures 1A, B**, significant differences were found between the 3 groups. Expression of the DNA repair genes in the normal group was significantly higher than that in the other 2 groups (p < 0.001). Moreover, expression of the DNA repair genes in the high-grade group was higher than that in the low-grade group (p = 0.0057). We assessed each DNA repair gene using ANOVA and ultimately obtained 120 differentially expressed genes (FDR < 0.05) (**Supplementary S3**). Further cluster analysis of these 120 gene expression profiles showed different expression patterns for these 120 genes in the 3 groups (**Figure 1C**).

### The Relationship Between Different Expression Patterns of DNA Repair Genes and Prognosis in Cancer Samples

We quantified the UCSC HT_HG-U133A chip data, containing 565 ovarian cancer cases with follow-up information, and further extracted 102 gene expression profiles for the abovementioned 120 genes.

Hierarchical clustering was used to cluster the expression profiles of DNA repair genes for each sample. The samples were divided into 3 clusters, as shown in **Supplementary S4a**. Only 1 sample was included in Cluster 3. The overall expression level of these 102 DNA repair genes in Cluster 2 was significantly higher than that in Cluster 1, and a significant prognostic difference was identified between Cluster 1 and Cluster 2, indicating that DNA repair was more active in the sample with the better prognosis than in the sample with the poorer prognosis (p = 0.026) (**Supplementary S4b**). A total of 28 DNA repair genes related to prognosis were obtained by univariate survival analysis. The most significant top 20 genes are shown in **Supplementary S5**, with a hazard ratio <1, indicating that lower expression of these genes was related to poor prognosis.

**Figure 2:**
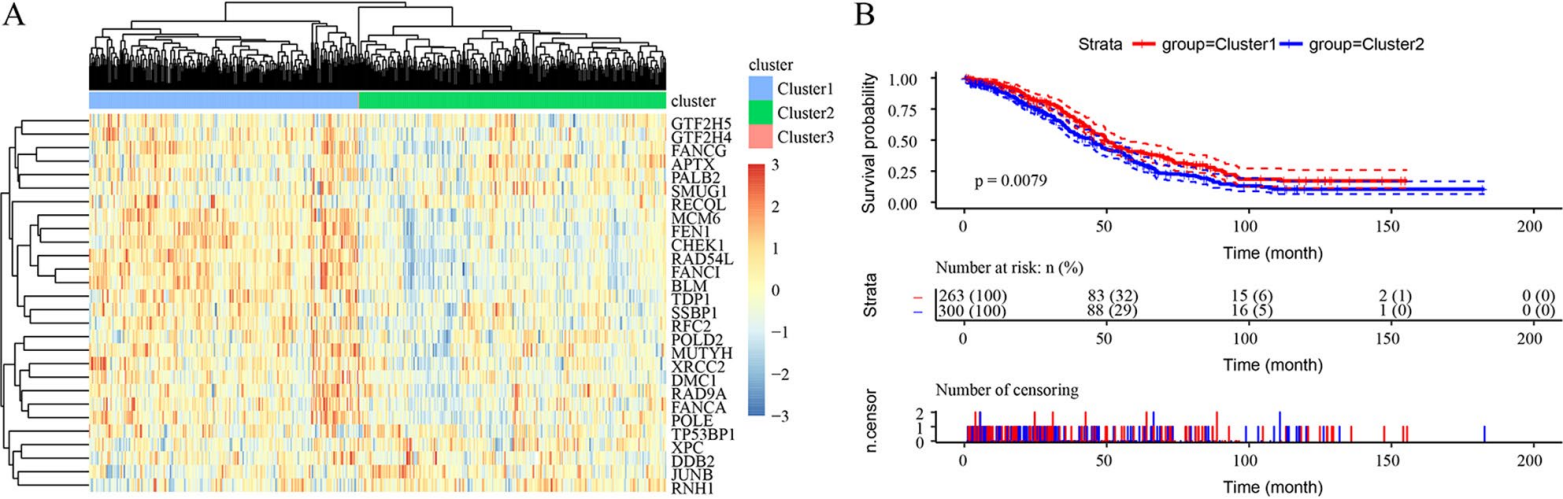
Unsupervised clustering based on 28 prognostic DNA repair genes from the TCGA HT_HG-U133A data set. **(A)** Heatmaps of 3 clusters divided by the expression of 28 prognostic DNA repair genes. **(B)** The prediction of prognosis between different clusters.

Unsupervised clustering based on these 28 prognostic DNA repair genes is shown in **Figure 2A**. Twenty-eight genes divided the samples into 3 clusters, though Cluster 3 contained only 1 sample. Expression of DNA repair genes in the Cluster 2 and Cluster 1 samples was significantly different, as was the prognosis of the 2 groups (p = 0.0079) (**Figure 2B**).

### Prognostic Signature Identification for Ovarian Cancer Patients

**Figure 3:**
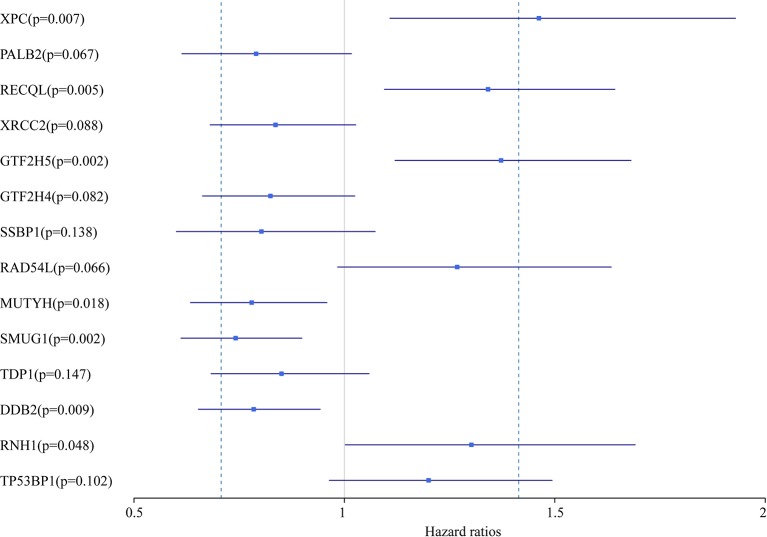
The forest map of 14 DNA repair genes in the prognostic model.

**Figure 4:**
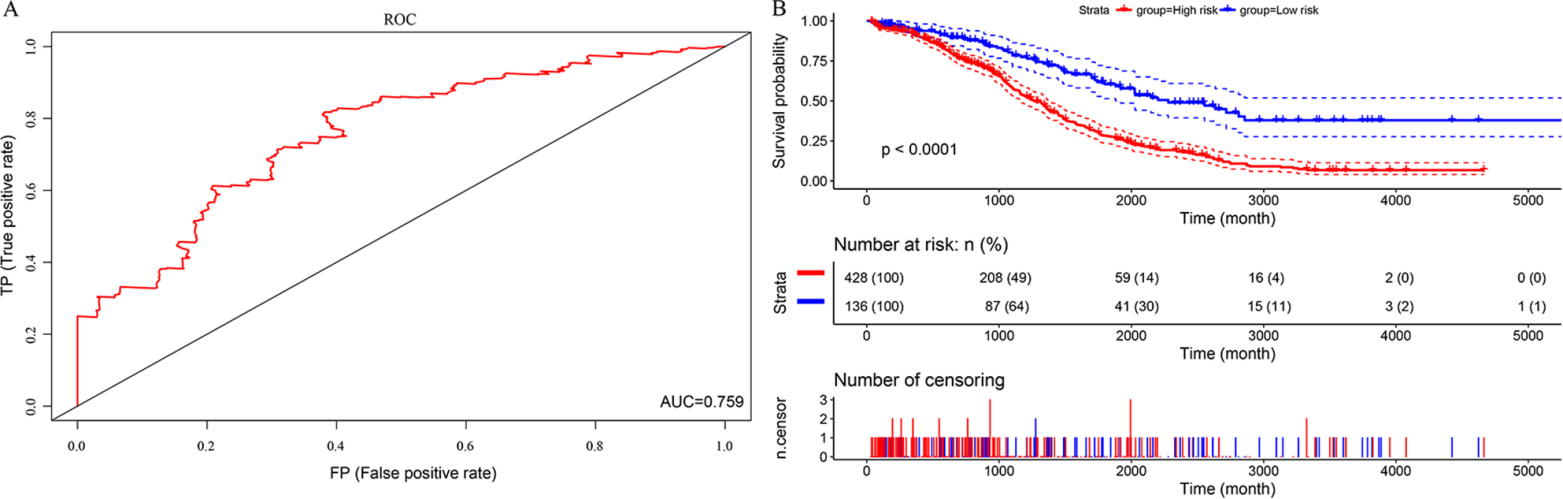
The predictive performances of the prognostic model based on the TCGA HT_HG-U133A data set. **(A)** ROC analysis of the prognostic model. **(B)** Kaplan-Meier survival analysis of the prognostic model.

In this study, we aimed to obtain a prognostic signature for ovarian cancer prediction. Therefore, we selected the 28 DNA repair genes for constructing a proportional hazard model by multivariate regression and a prognostic signature of DNA repair genes by stepwise regression. A total of 14 DNA repair genes were included. The risk model was as follows: Risk Score = 0.38*XPC – 0.24*PALB2 + 0.29*RECQL – 0.18*XRCC2 + 0.32*GTF2H5 – 0.19*GTF2H4 – 0.22*SSBP1 + 0.24*RAD54L – 0.25*MUTYH – 0.3*SMUG1 – 0.16*TDP1 – 0.24*DDB2 + 0.26*RNH1 + 0.18* TP53BP1. The forest map of these genes is shown in **Figure 3**. Eight genes had a hazard ratio <1, and 6 genes had a hazard ratio >1. To observe the stability of the model, we performed ROC analysis; as shown in **Figure 4A**, the AUC was 0.759. Kaplan-Meier survival analysis of the 2 groups after classifying the samples using the optimal threshold of 0.078 showed a significant difference in prognosis (p < 0.0001) (**Figure 4B**).

### Prognostic Signature Evaluation

To verify that these 14 genes were reproducible and portable in relation to ovarian cancer prognosis, we obtained another RNA-Seq dataset from TCGA containing 364 samples of cancer patients with follow-up information as an independent validation dataset for survival analysis. Expression of these 14 DNA repair genes in the 364 samples was extracted for multivariate analysis with the Cox regression model, and the ROC curves, with AUC values of 0.76, are shown in **Supplementary S6a**. Further analysis of prognosis after classification of the samples is shown in **Supplementary S6b**. The 14 genes had a significant classification effect on ovarian cancer prognosis in the external data, as the survival of the low-risk group was significantly better than that of the high-risk group (p < 0.0001), further demonstrating that the 14 DNA repair genes that we screened are key genes related to the prognosis of ovarian cancer.

### Evaluation of the Prognosis of Ovarian Cancer and DNA Repair Genes by IHC

**Figure 5:**
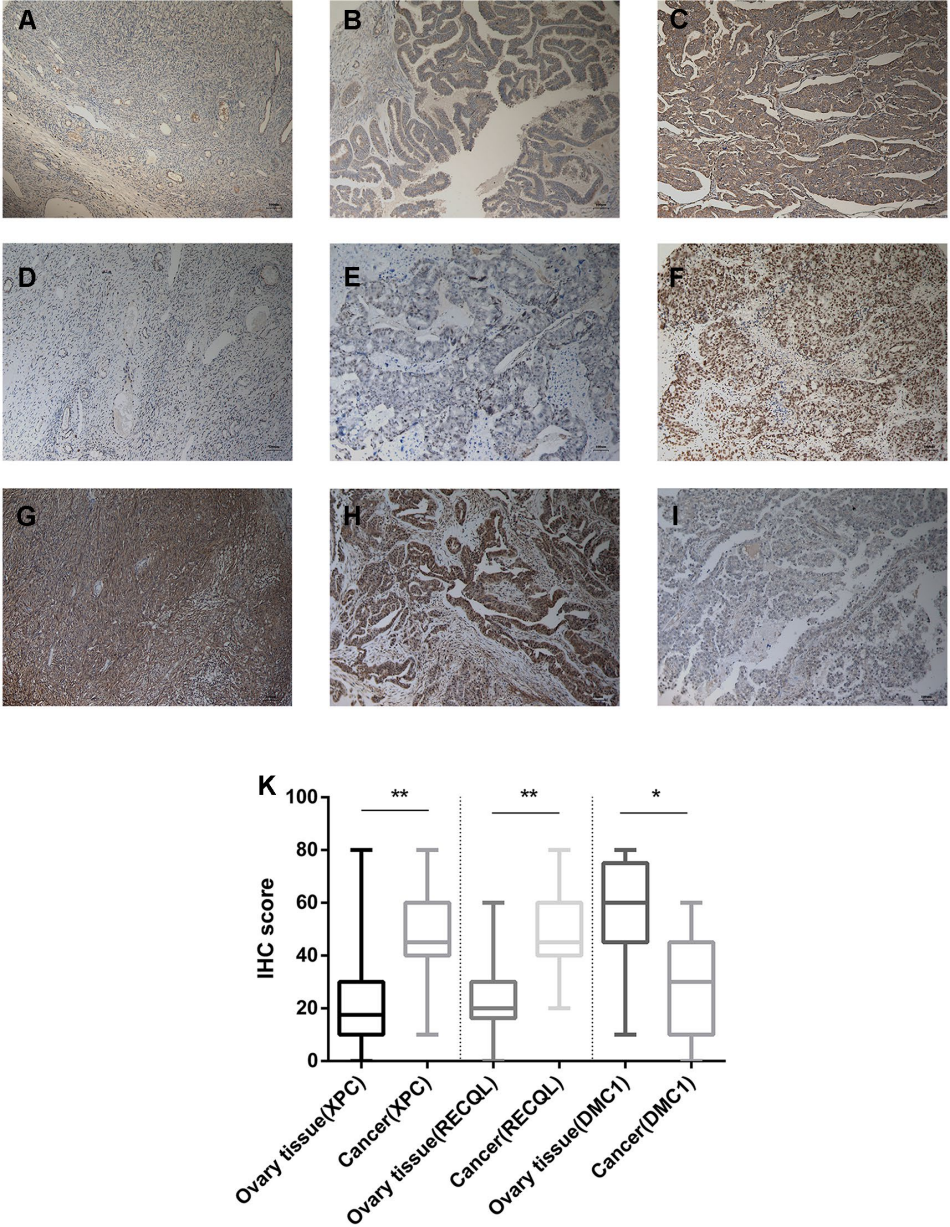
Immunohistochemistry for *XPC*, *RECQL*, and *DMC1*. Samples of ovarian tissue (N = 40) and ovarian cancer (N = 200). Cancer-adjacent ovarian tissue samples with weak immunostaining scores for **(A)** *XPC* or **(D)** *RECQL* or strong immunostaining scores for **(G)** *DMC1*. Ovarian cancer sample of weak and strong immunostaining score for *XPC* **(B**, **C)** or *RECQL* **(E**, **F)** and *DMC1* **(I**, **H)**, respectively. Expression of the *XPC*, *RECQL*, and *DMC1* genes is depicted in **(K)** slides (X 100). *p < 0.05, **p < 0.01.

**Figure 6:**
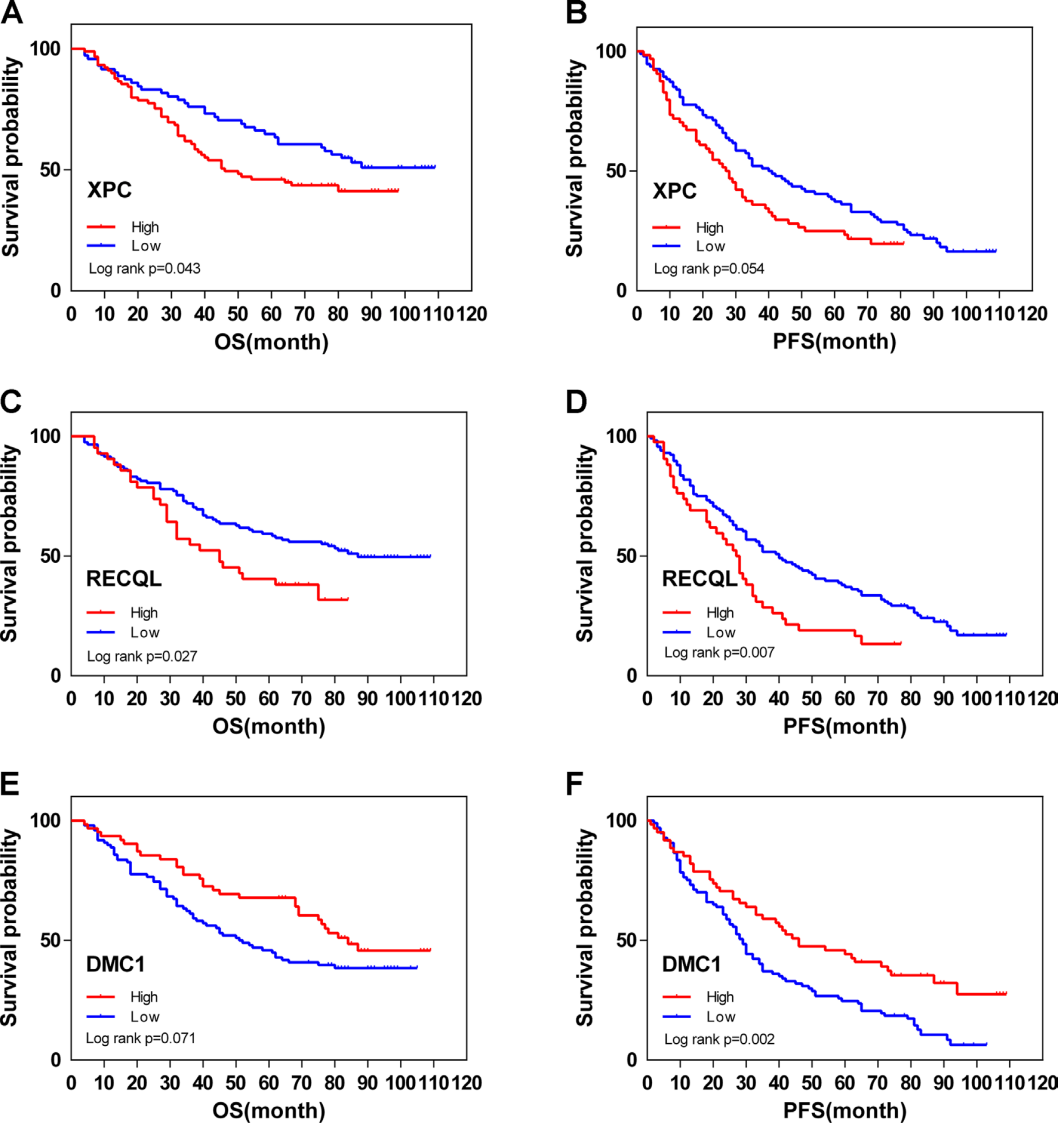
Overall (OS) and disease-free (DFS) survival curves for ovarian cancer (N = 160) according to *XPC* **(A**, **B)**, *RECQL* **(C**, **D)**, and *DMC1* **(E**, **F)** gene expression status (low or high). Gene expression status was divided according to their median values.

From February 2009 to February 2013, 200 human ovarian tissue samples, 160 of which had accompanying follow-up information, and 40 cancer-adjacent ovarian tissue samples from archives of paraffin-embedded tissues were collected at the Department of Pathology of Peking Union Medical College Hospital. The follow-up was performed until March 30, 2018. **Supplementary S7** summarizes the characteristics of all patients, including age, disease stage, and tumor grade. We selected the top 3 genes (*XPC*, *RECQL*, and *DMC1*, excluding *SUMG1* and *GTF2H5*, which have been evaluated in previous studies of ovarian cancer) that have rarely been studied in ovarian cancer to evaluate gene expression values by IHC. The differences in *XPC*, *RECQL*, and *DMC1* expression between ovarian cancer tissues and adjacent normal ovarian tissues are shown in **Figure 5**. Expression of *XPC* (50.18 ± 1.2 vs 23.13 ± 2.8, p < 0.01) and *RECQL* (46.20 ± 1.0 vs 25.25 ± 2.3, p < 0.01) was significantly higher in ovarian cancer than in adjacent cancer tissue. Conversely, *DMC1* (28.28 ± 1.5 vs 57.63 ± 2.7, p < 0.05) showed lower expression in ovarian cancer tissue. In addition, the correlation between expression of these genes and ovarian cancer prognosis is shown in **Figure 6**. These data reveal that high expression of *XPC* (OS, HR = 1.473, 95% CI 1.032–2.264, p = 0.043; PFS, HR = 1.403, 95% CI 1.005–2.114, p = 0.053) and *RECQL* (OS, HR = 1.658, 95% CI 1.085–3.032, p = 0.027; PFS, HR = 1.668, 95% CI 1.201–2.906, p = 0.007) and low expression of *DMC1* (OS, HR = 1.483, 95% CI 0.9710–2.225, p = 0.071; PFS, HR = 1.762, 95% CI 1.233–2.479, p = 0.002) are associated with poor prognosis in patients with ovarian cancer.

## Discussion

Approximately 151,900 women die from ovarian cancer every year worldwide due to the advanced stage of the disease at primary diagnosis and subsequent chemoresistance (Torre et al., 2015). Therefore, effective prognostic and therapeutic strategies to reduce the mortality rate of ovarian cancer are being actively explored. Together with an increased understanding of the role of DNA repair systems, targeting DNA repair/DDR defects through synthetic lethality has provided a paradigm for advanced clinical strategies (Kaelin, 2005; Oza et al., 2015). Approximately 50% of high-grade serous ovarian cancers are characterized by HR deficiency, which involves mutations in *BRCA1/2*, the *MRN* complex (*MRE11*, *RAD50*, *NBS1*), *ATM*, *RAD51C/D*, *PALB2*, *BRIP1*, *BARD1*, and other genes (O’Connor, 2015; Moschetta et al., 2016). It has also been reported that DNA repair/DDR defects are associated with the prognosis of ovarian cancer (Konstantinopoulos et al., 2015). In the present study, we used a high-throughput (based on TCGA and GEO data) method to search for genetic differences in terms of DNA repair genes associated with ovarian cancer prognosis and conducted a comprehensive analysis to obtain more reliable targets of DNA repair genes and a prognostic signature for ovarian cancer survival prediction. Ovarian cancer is a highly heterogeneous tumor with a wide variety of types, of which ovarian epithelial cancer is the most common, accounting for 80–90% of ovarian cancer cases. Ovarian cancer is divided into various types according to histological and pathological morphological differences, as follows: serous carcinoma, mucinous carcinoma, endometrioid carcinoma, clear cell carcinoma, and other types of tumors. In 2004, Professor Kurman, proposed a binary model theory based on a series of morphological and molecular genetics data: type I and type II (Shih Ie and Kurman, 2004). Ovarian serous carcinoma is divided into “high-grade serous carcinoma” and “low-grade serous carcinoma.” The 2 types of ovarian cancer have obvious clinical pathological and molecular differences. Once it seemed obvious that ovarian cancers, including high-grade serous ovarian cancer (HGSC), originated in the ovarian surface epithelium (Bell, 2005). In some patients, ovarian cancer is confined to the ovary. Though observed in different patients, viewing these tumors as different phases of the same malignancy, ovarian cancer with advanced-stage disease was assumed to have originated in the ovary (Scully, 1995; Feeley and Wells, 2001). Most advanced-stage ovarian cancers are high-grade serous carcinoma (HGSC). Hence, HGSC is also thought to arise from the ovary (Bell, 2005; Scott and McCluggage, 2006). Moreover, Ovarian carcinomas histopathologically resembling human HGSC can also arise from the ovarian surface epithelium (Flesken-Nikitin et al., 2003; Szabova et al., 2012; Tanwar et al., 2014). Mutations in the p53 gene (TP53 in humans; Trp53 in mice is the most common genetic event observed in human HGSC (2011). At present, it seems that many HGSC arises from serous tubal intraepithelial carcinoma (STIC) formed in the distal fallopian tube epithelium, however, logical and intuitive as it may seem, many of precursor or premalignant lesions, despite consisting of microscopically and genetically cancerous cells, would not progress to lethal malignancies (Esserman et al., 2013; Nikiforov et al., 2016; Brawley, 2017). Mouse studies suggest that many STIC lesions may not progress to invasive, and more critically, metastatic malignancies (Powell et al., 2011; Perets et al., 2013; Zhai et al., 2017). Studies of human HGSCs also note that most STIC lesions likely do not advance to metastatic HGSC, and may thus be classified as low grade (Carlson et al., 2008; Jarboe et al., 2008). Hence, it remains to be elucidated whether STIC could be a bona fide precursor lesion for HGSC in women in the general population who are at average risk, and yet who account for most cases of HGSC. Also, STIC lesions may not be unique to HGSC. Though STICs are associated chiefly with HGSC, they are not exclusive to HGSCs of the ovary, fallopian tube, and peritoneum. These findings suggest that a significant number of HGSCs may derive from precursors independent of STICs (Lim and Oliva, 2013; Howitt et al., 2015; Kim et al., 2018). The overall fraction of ovarian cancers that originate in the fallopian tube is not known. Therefore, both may be the cell of origin in different fractions of cases. In present study, using this dataset in the analysis, we found a high proportion of abnormal expression of DNA repair genes in ovarian cancer (17%). Based on this finding, we speculated that DNA repair genes may have potential as prognostic markers for ovarian cancer, and thus analysis of subsequent large sample queues. In fact, most of the current research is compared the ovarian cancer and adjacent tissues so that to obtain differential expression genes. We will pay more attention to the study of the fallopian tubes in the future. In the present study, expression of DNA repair genes in the normal group was significantly higher than that in the other 2 groups, and DNA repair gene expression in the high-grade group was also higher than that in the low-grade group, demonstrating the differences in gene expression between “high-grade serous carcinoma” and “low-grade serous carcinoma.” In the present study, 32 ovarian cancer samples and 15 matched para-carcinoma samples were analyzed as a training set to identify potential survival-related biomarkers. Another dataset containing 565 ovarian cancer samples with follow-up information was used to re-screen biomarkers related to survival. After hierarchical clustering and univariate survival analysis, 28 survival-related DNA repair genes were retained. We also constructed a proportional hazard model and a prognostic signature of DNA repair genes by stepwise regression based on 14 of the 28 genes. Ultimately, 5 genes showed significance in univariate analysis.

*XPC*, *SMUG1*, and *GTF2H5* were the top 3 most significant genes associated with ovarian cancer survival according to the prognostic signature. *XPC* plays a central role in the early steps of global genome nucleotide excision repair (NER), including damage sensing and DNA binding, and shows a preference for single-stranded DNA. Mutations in XPC can result in a rare autosomal recessive disorder termed Xeroderma pigmentosum, which is characterized by increased sensitivity to sunlight and the development of carcinomas at an early age (Sugasawa, 2016). Recently, *XPC* polymorphisms have been demonstrated to be associated with an increased risk for several types of human malignancies, such as lung, bladder, breast, and esophageal cancers (Zhu et al., 2008). In addition, the *XPC* rs2228001 A>C polymorphism has a significant association with an increased risk of ovarian cancer, whereas the variant rs2228000 C>T has the opposite association (Zhao et al., 2018). Zhao et al. also reported 3 intronic *XPC* SNPs (*XPC*-PAT, rs3731108 and rs1124303) to be associated with prolonged PFS in ovarian cancer, possibly suggesting improved platinum sensitivity (Fleming et al., 2012). However, these authors did not measure mRNA or protein expression of *XPC* to validate their findings. Therefore, we evaluated the correlation between *XPC* expression and the prognosis of ovarian cancer by IHC, showing that higher expression of *XPC* was associated with a poor prognosis. Therefore, we believe that *XPC* plays a crucial role in the DNA repair pathway of ovarian cancer. *SMUG1* is a key enzyme involved in BER that functions by removing uracil from single- and double-stranded DNA and is always associated with rectosigmoid junction neoplasms and bone lymphoma. In addition, increased expression of *SMUG1* is often found in various tumor types (including bladder, gastric, breast, esophageal, and cervical cancers) with adverse clinicopathological features, such as poorly differentiated and chemoradiotherapy-resistant tumors (Abdel-Fatah et al., 2013; An et al., 2013; Korourian et al., 2017). Further study has shown increased survival in colorectal cancer for carriers of the genotype *SMUG1* rs2233921 TT compared with those with the GT/GG genotypes (Pardini et al., 2013). GTF2H5 participates in the general and transcription-coupled NER of damaged DNA by opening the DNA around a lesion and initiating RNA transcription. Gayarre J et al. evaluated the prognostic and predictive value of GTF2H5 based on IHC staining in 139 ovarian cancer samples and demonstrated that low GTF2H5 expression is associated with an improved prognosis in ovarian cancer patients, which may be due to cisplatin sensitization (Gayarre et al., 2016).

In addition to the abovementioned genes, many cancer susceptibility, progression, and chemotherapy resistance-related DNA repair genes, such as *RECQL* and *DMC1*, were included among the 28 genes in our univariate survival analysis. *RECQL* is involved in various types of DNA repair, including mismatch repair, NER, and direct repair. *RECQL* has been found to be overexpressed in many other tumors and plays a critical role in malignant progression and PARPi resistance and may serve as a potential prognostic or predictive factor for ovarian cancer (Li et al., 2016; Vittori et al., 2017; Viziteu et al., 2017). *DMC1* has been reported to be an essential recombinase for meiotic homologous recombination and plays an important role in generating the diversity of genetic information. Loss of *DMC1* expression is found in multiple human cancers, and SNPs for *DMC1* are associated with cervical cancer. More importantly, *DMC1* interacts directly with the DNA repair gene *BRCA2*, which may provide possibilities for synthetic lethality targets for ovarian cancer (Harada et al., 2001; Martinez et al., 2016). In the present study, we evaluated expression of *RECQL* and *DMC1* in ovarian cancer samples and found that higher expression of *RECQL* and lower expression of *DMC1* were associated with poor prognosis, suggesting that *DMC1* deficits contribute to the progression of ovarian cancer and subsequently enhance other DNA repair genes, such as *XPC* and *RECQL*.

In recent years, many prognostic signatures based on oxidative stress- and immunogenomic-related genes, alternative splicing, genomic and epigenomic mechanisms, DNA methylation, miRNA, and long non-coding RNAs have been constructed, and the predictive performances of these models in chemotherapy-sensitive ovarian cancer have been validated. Nonetheless, few DNA repair gene-related models have been constructed to explore the prognosis of ovarian cancer. Notably, Kang et al. hypothesized a DNA repair pathway-focused score for predicting outcomes and sensitivity to platinum-based chemotherapy in ovarian cancer; however, the accuracy was not high, as the area under the ROC curve was only 0.65, and no evaluation in ovarian cancer tissue was performed (Kang and D’Andrea, 2012). Wang et al. recently stratified ovarian cancer into 7 subgroups within histotypes based on their diverse DNA repair deficiency-related signatures, which were characterized by mutation signatures associated with mismatch repair deficiency, the AID/APOBEC family of cytidine deaminases, age at diagnosis, the prevalence of foldback inversion structural variations, the prevalence of duplications or deletion rearrangements and homologous recombination deficiency. Among the signatures, the prevalence of foldback inversion structural variations was also identified as a prognostically significant high-grade serous cancer group associated with poor survival compared with homologous recombination deficiency (Wang et al., 2017). Furthermore, Manuela Tumiati et al. developed the HR score, which is calculated as the percentage of RAD51-positive cells also positive for both cyclinA2 and CK7, to predict platinum sensitivity and overall survival. The authors reported that low HR scores correlated with platinum sensitivity and improved overall survival (Tumiati et al., 2018). To the best of our knowledge, we herein present is the first high-accuracy prognostic model of ovarian cancer constructed using multiple DNA repair genes. The 14 DNA repair genes in the signature that were evaluated not only show high prediction accuracy but also provide potential targets, including but not limited to *BRAC1/2*, for synthetic lethality. Although the 14-gene signature has significant prognostic value, some limitations remain; for example, we did not evaluate the synergistic effect of each DNA repair gene in ovarian cancer due to a lack of data. We will further evaluate the synergistic effect of the DNA repair genes in the signature.

## Conclusion

In conclusion, our study profiled DNA repair genes that are consistently altered between ovarian cancer and normal control samples from the GEO and TCGA databases, and these genes were also evaluated in terms of prognostic prediction based on our samples. The combination of these biomarkers may serve as a signature to stratify ovarian cancer patients into low-risk and high-risk groups for assessing overall survival, which should be helpful for precision and personalized treatment.

## Data Availability

All datasets analyzed for this study are cited in the manuscript and the **Supplementary files**.

## Ethics Statement

All procedures performed in studies involving human participants were in accordance with the ethical standards of the institutional and national research committee and with the 1964 Helsinki declaration and its later amendments. The project was approved by the institutional ethics committee of Peking Union Medical College Hospital, CAMS Chinese Academy of Medical Sciences, No. S-825 2018. Informed consent was obtained from all participants included in the study.

## Author Contributions

XH: Study design and data analysis, KS: Study design, funding and clinical data provider, HS: Data collection, data analysis, manuscript writing, follow-up, DC, XM, JY, PP, MY, HZ, YZ, LL: Clinical data provider. All authors have read, edited, and approved the final version of the manuscript.

## Funding

This study was funded by the Beijing Science and Technology Plan Project [D151100001915004] (KS) and [CAMS Initiative for Innovative Medicine CAMS-2017-I2M-1-002] (KS).

## Conflict of Interest Statement

The authors declare that the research was conducted in the absence of any commercial or financial relationships that could be construed as a potential conflict of interest.
