# Supplementary material for: Identification of a Prognostic Signature Associated With DNA Repair Genes in Ovarian Cancer
Source: Front Genet. 2019 Sep 12;10:839. doi: 10.3389/fgene.2019.00839 (PMC6751318; doi:10.3389/fgene.2019.00839)

**Supplementary S1.** Data standardization of GSE14001 and GSE14407. (a) Chip raw data after RMA standardization. (b) Expression boxplot after removing the batch effect. (c) The quantile normalization result after the batch effect has been removed.


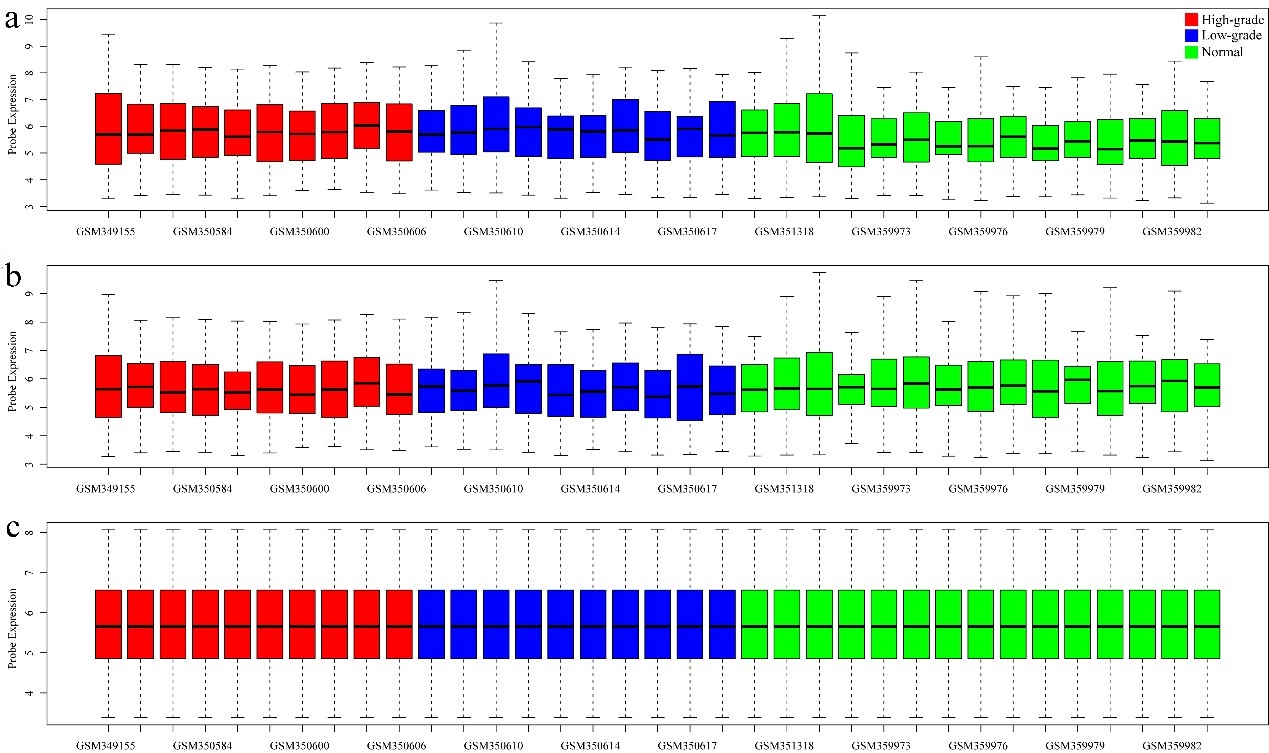

Supplement: Supplementary file 1 [file Table_1.docx]
