# Supplementary material for: Identification of a Prognostic Signature Associated With DNA Repair Genes in Ovarian Cancer
Source: Front Genet. 2019 Sep 12;10:839. doi: 10.3389/fgene.2019.00839 (PMC6751318; doi:10.3389/fgene.2019.00839)
Supplement: Supplementary file 2 [file Table_2.docx]

**Supplementary S2.** The 727 DNA repair genes were obtained from the DNA repair-related pathways in the KEGG and the literature.

| **Gene** | **Gene** | **Gene** | **Gene** | **Gene** | **Gene** |
| --- | --- | --- | --- | --- | --- |
| C17orf70 | p193 | FANCH | APTX | TDP1 | T-BTF2P44 |
| RPA32 | DCLRE1A | MTMR15 | ERCC5-201 | FA-H | GTF2H2 |
| hExoI | BLAP18 | hTDG | ERCC5-202 | MYH | LIG4S |
| RPRGL4 | P52 | PARP-2 | NTH1 | DLEU8 | GTF2H5 |
| UBE2V2 | GTF2H2C_2 | PARP-1 | TP53 | LIG1 | GTF2H4 |
| COCA2 | PPP1R53 | PARP-4 | BTF2 | LIG2 | REPA2 |
| COCA1 | MDC1 | p58 | MITOTIN | LIG3 | SSBP1 |
| HYRC1 | VPARP | UNG | UBE2A | NEI1 | SPG60 |
| DNA-PKcs | XPE-BF | DUP | PALB2 | NEI2 | NKCD |
| XPAC | CHRAC17 | HAP1 | VWA5C | NEI3 | PNAS146 |
| RNASEHI | NBN | DUT | CAP35 | APEN | C6orf175 |
| PRIM1 | hFPG2 | SYCP3 | POLE4 | HCAK | RNF168 |
| PRIM2 | GIYD1 | pADPRT-1 | POLE3 | APE1 | NFIV |
| XP3 | SEM1 | P1CDC47 | POLE2 | APE2 | REPA3 |
| XP1 | DSS1 | HsT16930 | POLE1 | CEN2 | RAD3OB |
| MUS312 | XPCE | pADPRT-2 | HOGG1 | RFC40 | REPA1 |
| XPC | ARTD4 | APNG | HLTF | MGMT | HHL |
| XPB | ARTD2 | APEXL2 | TOP3A | PMS1 |  |
| XPA | ARTD3 | RAD52 | AT-V2 | PMS2 |  |
| RNASEH1 | XPCC | RAD51 | AT-V1 | ML8 |  |
| XPG | ARTD1 | CDK7 | TOP3B | TDP2 |  |
| XPF | MO15 | XAB2 | MAD2L2 | CENP-S |  |
| XPE | CALT | Shfdg1 | p125 | UBP |  |
| XPD | MBD4 | ERCC8 | TEL2 | FA |  |
| STK1 | LCFS2 | POLA2 | ADPRT | OF |  |
| HSPC150 | UDG | AAG | FAP3 | CENP-X |  |
| XPV | DINB1 | UVSSA | POLA1 | H2AFX |  |
| SHFM1 | RP-A_p14 | ERCC1 | AGS4 | SPO11 |  |
| SPGF4 | G22P1 | ERCC2 | ATLD | P1-CDC46 |  |
| PIG11 | FILS | ERCC3 | SNM1C | RNF8 |  |
| PIG16 | KIAA1018 | ERCC4 | MST075 | RNF4 |  |
| HSU24186 | RAD10 | ERCC5 | BTBD12 | CCNH |  |
| TLAA | RAD17 | ERCC6 | HNPCC2 | RLFB |  |
| NEH2 | Mt-SSB | XAP1 | VAULT3 | RAD26 |  |
| NTHL1 | PADPRT-3 | p350 | HNPCC1 | D3S3194 |  |
| FGP2 | RAD18 | gs125 | HNPCC7 | MAT1 |  |
| P1-CDC21 | FAAP90 | MDPL | HNPCC4 | FEN-1 |  |
| PMSL2 | NSX | RAD54A | HNPCC5 | FEN1 |  |
| HMUDG | SLX1A | RAD54B | ATLD2 | RNH1 |  |
| RAD4 | HIGM4 | hMLH1 | GTMBP | LIG4 |  |
| RAD2 | SLX1B | RAD54L | SCKL1 | POLM |  |
| RAD1 | hRad50 | DNPK1 | BTF2P44 | MMS19 |  |
| MRP1 | CCNL1 | FCTCS | hFPG1 | POLL |  |
| FA-D2 | FAD2 | POG | USP1 | RECQ2 |  |
| HR23A | FAD1 | CDC54 | Mis5 | CRCS10 |  |
| HR23B | HRY | hNEI3 | MSH2 | CRCS12 |  |
| GLM3 | RNF66 | BLAP75 | P34 | UV20 |  |
| SLX2A | KARP-1 | FAAP250 | MSH3 | GTF2H3 |  |
| WDR48 | p12 | BETAN | MSH4 | HYRC |  |
| BACH1 | REV3L | TTD-A | PMS2L3 | UV-DDB2 |  |
| CETN2 | p17 | BROVCA4 | MSH5 | UV-DDB1 |  |
| hFAN1 | CENPS | BROVCA2 | HSSB | NBS1 |  |
| GTF2H | NHEJ1 | CUL-4B | YBL1 | GTF2H1 |  |
| TTD | SBP-1 | P1.1-MCM3 | TGF2H5 | p160 |  |
| XFEPS | APX | BROVCA1 | MSH6 | hCdc21 |  |
| RAD30B | ADPRT_1 | ENDOV | MRMV2 | MRXHF2 |  |
| SPRTN | COR1 | ZGRF2 | RAD51D | UNG1 |  |
| RAD30A | OBFC2B | ZGRF3 | RAD51C | UNG2 |  |
| BRCC5 | APE | P62 | RAD51B | UNG3 |  |
| cdc19 | RECQL5 | p180 | RAD51A | p39MO15 |  |
| BRCC2 | RECQL4 | TOPBP1 | HNGS1 | RECQL |  |
| BRCC1 | P80 | P66 | TRAD | GIYD2 |  |
| BLM | PPOL | FACB | PSCP | TTDN1 |  |
| CTC75 | RECQL3 | FACC | UBE2N | p37 |  |
| HMMH | RECQL2 | FACA | TDG | p34 |  |
| CSB | XLF | FACD | OCTS3 | MPG |  |
| HMG-1 | MED1 | FACE | C9orf76 | anpg |  |
| hRAD54 | DINP | CUL4A | RP-A_p32 | RNHL |  |
| CDKN7 | SHPRH | CMM6 | FAAP16 | CDC21 |  |
| TOP3 | TREX2 | CUL4B | RP-A_p34 | TFIIH |  |
| APITD1 | EXO1 | UVSS1 | FAAP10 | RDH54 |  |
| TFB1 | P95 | UVSS2 | Ku86 | BROVCA3 |  |
| TFB2 | AYP1 | D9 | TDT | RAD23B |  |
| TFB3 | ATRIP | ADPRTL1 | UBE2B | RAD23A |  |
| TFB4 | DPE2 | ADPRTL2 | RAD30 | RAD51L1 |  |
| TFB5 | HCC5 | ADPRTL3 | KUB2 | RAD51L2 |  |
| SOSS-B1 | MRE11 | XP-V | RECA | RAD51L3 |  |
| DNA2L | CSA | MHCBFB | ERCC11 | MUTYH |  |
| TTD1 | XTH2 | RAD9A | DCLRE1B | UBE2T |  |
| TTD2 | CRA36.1 | CDCL1 | DCLRE1C | MHF1 |  |
| TTD3 | RFC | Mid1 | RCC | CycH |  |
| POLQ | ECD | FAAP43 | DNA2 | MHF2 |  |
| hNTH1 | SSMED | ERCM2 | SHSF1 | KU80 |  |
| RNF53 | BIVM-ERCC5 | Fanconi | NUDT1 | P1-MCM3 |  |
| POLZ | TELO2 | KARP1 | APEX1 | POLKAPPA |  |
| PRKDC | H1RNA | PARP | FAH | XRCC11 |  |
| POLG | SLX4 | KMIN | APEX2 | TREX1 |  |
| MUS81 | PEOB2 | HSAP | DMC1 | NBSLD |  |
| POLE | RAD502 | BRCAI | FAB | NBS |  |
| POLD | RNASEH2A | RS-SCID | FAC | HUS1 |  |
| POLB | RNASEH2C | PPP1R104 | FAA | P1.h |  |
| p49 | RNASEH2B | BRCA1 | FAF | POLD1 |  |
| RP-A | hHR54 | BRCA2 | FAG | POLD2 |  |
| POLN | XPGC | PNKP | FAD | POLD3 |  |
| CDC2 | NER-related | PER1 | FAE | POLD4 |  |
| bHLHb39 | FAAP75 | GTBP | PCNA | KU70 |  |
| POLK | MCM4 | KIAA1794 | POL4P | COFS1 |  |
| POLI | NEIL1 | MRXS15 | PH5P | COFS2 |  |
| POLH | NEIL3 | SMUG1 | FRP1 | COFS3 |  |
| mtSSB | NEIL2 | RBBP8 | FA2 | COFS4 |  |
| PO-GA | MCM3 | SFM2 | FA3 | SETMAR |  |
| REF1 | CLK2 | COFS | CENPX | APLF |  |
| MDG | ALKBH2 | IMD26 | FA1 | CAK1 |  |
| bA120J8.2 | ALKBH3 | RBX1 | ROC1 | DDB2 |  |
| RF-A | FAAP95 | RNHIA | FA4 | POLDS |  |
| CHARAC17 | BM28 | EME1 | MUTM | OGH1 |  |
| hDNA2 | C16orf75 | EME2 | MCM7 | HNPCC |  |
| DDB1 | RFC5 | PMS2CL | MCM6 | MNAT1 |  |
| RIF1 | RFC4 | HELQ | MCM5 | ZGRF7 |  |
| DDBB | ARMD5 | SHFD1 | RECC1 | POLA |  |
| DDBA | ATM | FDG | TP53BP1 | HsRad51 |  |
| ADPG | RFC1 | RNF75 | MCM2 | PIG50 |  |
| PRIM2A | RFC3 | BS | HES1 | SCKL |  |
| TOP3B1 | RFC2 | XRCC9 | REV1L | HR54 |  |
| MEC1 | ATV | XRCC1 | EM9 | P68 |  |
| GEN1 | ATR | XRCC3 | RPA1 | FANCT |  |
| P85MCM | FANCD1 | XRCC2 | RPA3 | UAF1 |  |
| PPP1R128 | FANCD2 | XRCC5 | RPA2 | P105MCM |  |
| GTF2H2C | IRT1 | XRCC4 | A-SCID | JUNB |  |
| KIAA1596 | BRIP1 | XRCC7 | REC2 | SCP3 |  |
| GTF2H2D | MRE11B | XRCC6 | ADPRT3 | MMS4L |  |
| TTDA | SLX3 | HHR23B | ADPRT2 | STRA13 |  |
| SLX2B | MRE11A | HHR23A | ADPRT1 | RDM1 |  |
| RPA4 | FANCS | MCG40308 | UVDR | p44 |  |
| CHAF1A | FANCR | PARPL | RFC37 | RMI1 |  |
| CDC47 | FANCQ | RFC140 | SCIDA | RMI2 |  |
| CDC46 | FANCP | RRM2B | HRAD51 | PHF9 |  |
| RPA70 | DCLREC1C | CTCBF | HIGM5 | SSBP |  |
| YHHQ1 | FANCG | CHEK2 | R51H3 | RFC38 |  |
| PNCA3 | FANCF | CHEK1 | R51H2 | FCC1 |  |
| PNCA2 | FANCE | DNAPK | BA554C12.1 | FCC2 |  |
| HMGB1 | FANCD | PARP4 | HMG1 | PRPF19 |  |
| PNCA4 | FANCC | RAD50 | OGG1 | MLH4 |  |
| CAK | FANCB | NKGCD | HMG3 | MLH3 |  |
| MRXSC | FANCA | PARP1 | C19orf40 | RFC36 |  |
| UNG15 | DGU | PARP2 | FAAP100 | MLH1 |  |
| HEX1 | FANCO | PARP3 | DNTT | A1 |  |
| IRIS | FANCN | FAN1 | APEX | Pol_Mu |  |
| WRN | FANCM | MF1 | AGS3 | FAAP20 |  |
| P58 | FANCL | HES-1 | AGS2 | FAAP24 |  |
| FPG2 | FANCJ | REV1 | CKN1 | Tdt-N |  |
| FPG1 | FANCI | REV3 | CKN2 | RAD25 |  |
