# Supplementary material for: Identification of a Prognostic Signature Associated With DNA Repair Genes in Ovarian Cancer
Source: Front Genet. 2019 Sep 12;10:839. doi: 10.3389/fgene.2019.00839 (PMC6751318; doi:10.3389/fgene.2019.00839)
Supplement: Supplementary file 3 [file Table_3.docx]

**Supplementary S3.** The 120 DNA repair genes were identified by ANOVA analysis (FDR < 0.05).

| **Gene** | **p.value** | **HR** | **Low 95%CI** | **High 95%CI** |
| --- | --- | --- | --- | --- |
| XPC | 0.0417176686340935 | 1.27337301549614 | 1.00910371966893 | 1.60685051991059 |
| FANCI | 0.00116523701655602 | 0.816309480571042 | 0.72219184418297 | 0.92269273523025 |
| RAD9A | 0.0434900500075573 | 0.803014049418407 | 0.648979467336002 | 0.993608574721662 |
| CDK7 | 0.502839365092572 | 0.945306450993604 | 0.801895582116762 | 1.11436489515415 |
| TDG | 0.670130818676654 | 0.965760799009824 | 0.822719004083206 | 1.13367251306348 |
| RMI1 | 0.221343285492709 | 0.913605379683979 | 0.790440374445824 | 1.05596173572573 |
| FEN1 | 0.0111411586602641 | 0.800583153013691 | 0.674247544256175 | 0.950590610747298 |
| BRIP1 | 0.388982299318011 | 0.871348627879128 | 0.636970692290433 | 1.19196760619663 |
| XPA | 0.221476643887881 | 1.13953506557748 | 0.924246261328526 | 1.40497205129522 |
| HLTF | 0.655664455913252 | 0.963484999447186 | 0.818156141021113 | 1.13462858446697 |
| PALB2 | 0.00585819446819946 | 0.723119805644721 | 0.574211475335975 | 0.910644032339663 |
| PCNA | 0.266722233908895 | 0.909560350236196 | 0.769451091720619 | 1.07518208710551 |
| RPA1 | 0.856157951642065 | 1.01632002365037 | 0.853125373818046 | 1.21073223487663 |
| RNF4 | 0.346792429729729 | 0.91424444246154 | 0.758483580022796 | 1.1019920833971 |
| RECQL | 0.0326359829382772 | 1.23063588562349 | 1.01729887328582 | 1.48871164881239 |
| RECQL5 | 0.402168808031344 | 0.740235418897489 | 0.366230655730947 | 1.49618407638954 |
| RECQL4 | 0.129560129137776 | 0.834054873674849 | 0.659629517019107 | 1.05460340138268 |
| MUS81 | 0.128816112877815 | 0.826389976753198 | 0.646121226555916 | 1.05695396716556 |
| BRCA1 | 0.300437823397657 | 0.875020751757412 | 0.679628932206437 | 1.12658728862581 |
| BRCA2 | 0.130679753986012 | 1.27478035166845 | 0.930487555162683 | 1.74646607145199 |
| XRCC2 | 0.0263761352644518 | 0.820616228781871 | 0.689221335930517 | 0.97706057522292 |
| XRCC4 | 0.512273760189761 | 1.11777373789139 | 0.801180746697394 | 1.55947098612891 |
| POLH | 0.994872075690649 | 0.999360943992401 | 0.82235065871908 | 1.21447254378445 |
| RNASEH1 | 0.487340554368449 | 0.930004643098426 | 0.757795477923354 | 1.14134837351472 |
| MBD4 | 0.708809434773464 | 1.0429718190475 | 0.836324293911572 | 1.30067991955554 |
| CLK2 | 0.974201907597272 | 0.996512148222638 | 0.806337652973181 | 1.23153924152457 |
| LIG4 | 0.911896882468391 | 1.02012048951913 | 0.71680520637616 | 1.45178327930649 |
| LIG1 | 0.288303512077261 | 0.889052859718292 | 0.715579861541874 | 1.10457969802301 |
| PNKP | 0.675417826844984 | 1.03921830802762 | 0.867971829215735 | 1.24425085629289 |
| ERCC2 | 0.130998200623705 | 1.25397672155492 | 0.934815174531143 | 1.6821053626887 |
| HUS1 | 0.388789580313223 | 1.16286368462679 | 0.825089889475949 | 1.63891470041242 |
| GTF2H5 | 0.0457097755900329 | 1.20946813510459 | 1.00363299155988 | 1.45751801917135 |
| GTF2H4 | 0.000879742245687765 | 0.719154699235207 | 0.592188583373138 | 0.873342539780445 |
| ATR | 0.144191149486013 | 0.842847429706988 | 0.670032553993206 | 1.06023473864059 |
| MPG | 0.920359280540789 | 0.990515391225448 | 0.821726909429904 | 1.19397421332494 |
| FANCG | 0.000484208607033132 | 0.756680456976386 | 0.646990175963603 | 0.884967554750326 |
| FANCC | 0.471270898642429 | 0.890102688083691 | 0.648464563638508 | 1.22178271529341 |
| FANCA | 0.0249839825906868 | 0.540500812786515 | 0.315623678469311 | 0.925599530553879 |
| MSH3 | 0.615533957427257 | 1.06673119615666 | 0.829012331869705 | 1.37261582380497 |
| UBE2N | 0.441262703854839 | 1.08401936635 | 0.88279678988086 | 1.33110813280195 |
| UBE2A | 0.77760052246969 | 1.0288767188871 | 0.844445084621968 | 1.25358927649131 |
| TOP3A | 0.255546796660379 | 0.715734958055535 | 0.402132531038547 | 1.27389974857233 |
| TOP3B | 0.0967459713224074 | 0.595866216136395 | 0.323445120529949 | 1.0977335102504 |
| DMC1 | 0.0168312061776485 | 0.456223702368623 | 0.239730928018669 | 0.868223671944092 |
| MLH1 | 0.375252311608791 | 0.909111434233128 | 0.736447886741315 | 1.12225673361695 |
| SSBP1 | 0.00485544827722439 | 0.699580085353035 | 0.545582961318062 | 0.897044685266928 |
| FAH | 0.127443148998546 | 0.911092166162661 | 0.808287289839416 | 1.02697264410515 |
| MNAT1 | 0.372766595618055 | 0.921340254869357 | 0.769449617194637 | 1.10321435773488 |
| RAD1 | 0.130891667081176 | 0.856358989622789 | 0.700290627065183 | 1.04720910257093 |
| UBE2V2 | 0.481321572314237 | 1.07857504485714 | 0.873809638480426 | 1.33132443973911 |
| NEIL3 | 0.308965432321647 | 1.15979933838519 | 0.87167044456326 | 1.54316865245177 |
| RAD54L | 0.0277539713850808 | 0.806423519530539 | 0.665808362745329 | 0.976735843585023 |
| PARP3 | 0.46587466123882 | 0.908243451210572 | 0.701226576112827 | 1.1763760741067 |
| POLD2 | 0.00691153773551978 | 0.769290385779026 | 0.63596730261133 | 0.930563089048815 |
| POLD4 | 0.494452094085402 | 1.07292554660755 | 0.876753141682935 | 1.31299127865504 |
| ECD | 0.590141445531694 | 1.05248096560872 | 0.873738182241764 | 1.26778960274641 |
| CHEK2 | 0.192010632605599 | 0.903602215753098 | 0.775965785555643 | 1.0522332034643 |
| CHEK1 | 0.0226428850784683 | 0.85485278363643 | 0.747012063103211 | 0.978261687843702 |
| CCNH | 0.16077959111493 | 1.14224216576051 | 0.948505214959173 | 1.37555086114884 |
| NBN | 0.0513921009845906 | 1.19647376725275 | 0.998916605231652 | 1.43310209103191 |
| MSH2 | 0.600629205707462 | 0.9610491045107 | 0.828218976835886 | 1.11518258710925 |
| MSH6 | 0.290774556674955 | 0.8857748538451 | 0.707283164961218 | 1.10931113671754 |
| MCM6 | 0.028526445849787 | 0.852763118917787 | 0.739471927363196 | 0.983411147978875 |
| MCM4 | 0.231817628856315 | 0.907040285723511 | 0.772980328391637 | 1.06435060467483 |
| PMS1 | 0.0967746465421464 | 0.87472184891531 | 0.746903547269208 | 1.02441381590338 |
| BLM | 0.00726241557202467 | 0.818016003692338 | 0.706432062158014 | 0.947225102230862 |
| RBX1 | 0.348417627869302 | 0.901938647456102 | 0.726917373022544 | 1.11910012604653 |
| MUTYH | 0.0274383475383205 | 0.816591286581545 | 0.682018464378517 | 0.977717414041771 |
| WRN | 0.33939565849151 | 0.921653903367404 | 0.779609737842908 | 1.08957838308009 |
| POLQ | 0.179783785056428 | 0.839957436179809 | 0.651011735688337 | 1.08374159161321 |
| POLE | 0.0189975774049594 | 0.751938264489801 | 0.592543303881786 | 0.95421068789385 |
| POLM | 0.803068106920886 | 0.963472120993054 | 0.719158498192257 | 1.2907843406763 |
| POLI | 0.0525667955077297 | 0.85642829444085 | 0.732209615931846 | 1.00172055591678 |
| RFC5 | 0.118029952027155 | 0.884857838923906 | 0.759028827171269 | 1.03154632219049 |
| RFC4 | 0.0796638420286968 | 0.893086053562345 | 0.787005310278722 | 1.01346546033481 |
| RFC1 | 0.668963564099547 | 0.957684636220814 | 0.785502323510545 | 1.1676093564618 |
| RFC2 | 0.0492171480336363 | 0.842760126061061 | 0.710661257970627 | 0.999413746158949 |
| ERCC8 | 0.462760889610315 | 1.08151330171743 | 0.877397518981933 | 1.33311412043761 |
| ERCC4 | 0.367802614707765 | 1.15792198596344 | 0.841578081813257 | 1.5931775726486 |
| ERCC5 | 0.95261346334591 | 1.00489936864284 | 0.855294647013719 | 1.1806723503118 |
| ERCC6 | 0.865399634785971 | 1.03381646361838 | 0.703777792851646 | 1.51862774202895 |
| SMUG1 | 0.00222122866281238 | 0.757539508697661 | 0.634068571186309 | 0.90505370131218 |
| TDP1 | 0.00272216835216188 | 0.741544268862445 | 0.609850938829883 | 0.901675914015653 |
| RNASEH2A | 0.1817797799423 | 0.916598430356602 | 0.806612783692384 | 1.04158116449168 |
| XAB2 | 0.260002606797348 | 1.18351221043545 | 0.882772210302221 | 1.58670734749372 |
| JUNB | 0.0171795293793098 | 1.15757040926692 | 1.02630783667995 | 1.30562118354772 |
| DDB2 | 0.0363502028333188 | 0.834831785855321 | 0.704986652512559 | 0.988591923252007 |
| CCNL1 | 0.388149882304854 | 0.932566739538235 | 0.795825822311199 | 1.09280284618975 |
| EXO1 | 0.506534037233171 | 0.948510000202525 | 0.811521868007714 | 1.10862227618448 |
| TP53 | 0.879857967141455 | 0.991824583766056 | 0.891672847456294 | 1.10322525550598 |
| APTX | 0.0327750164596107 | 0.81509331511816 | 0.675596984020343 | 0.983392655776432 |
| GTF2H1 | 0.765039239099011 | 1.03528238517511 | 0.824715838326991 | 1.29961080804284 |
| RNH1 | 0.0328560696274859 | 1.29078898028297 | 1.02101690539929 | 1.63183996543952 |
| HAP1 | 0.732022682179565 | 0.85007542557989 | 0.335506493933897 | 2.15384274891921 |
| CUL4B | 0.132110279713235 | 0.847707161435204 | 0.683678533093679 | 1.05108965217439 |
| RAD51C | 0.938781109435545 | 0.993099882811446 | 0.832249151096008 | 1.18503861006202 |
| DCLRE1C | 0.0922949157864529 | 0.846359528740962 | 0.696961138262063 | 1.02778248680672 |
| TP53BP1 | 0.036387307364009 | 1.23539060854996 | 1.01348598308024 | 1.50588166109113 |
| RAD51 | 0.640539948618901 | 0.936771075672802 | 0.712142664697795 | 1.23225315897844 |
| POLE3 | 0.538010384466171 | 0.957579858410783 | 0.834182952828442 | 1.09923030928036 |
| POLE2 | 0.110831055982786 | 0.899988426322235 | 0.790648993876704 | 1.02444848951554 |
| RAD23B | 0.258241444861618 | 1.13236753143577 | 0.912837381482428 | 1.4046929412198 |
