# Supplementary material for: Identification of a Prognostic Signature Associated With DNA Repair Genes in Ovarian Cancer
Source: Front Genet. 2019 Sep 12;10:839. doi: 10.3389/fgene.2019.00839 (PMC6751318; doi:10.3389/fgene.2019.00839)

**Supplementary S4.** Hierarchical clustering of DNA repair genes from TCGA HT_HG-U133A data set. (a) Heatmaps of three clusters divided by the expression of 102 DNA repair genes. (b) The different prognosis between different cluster.


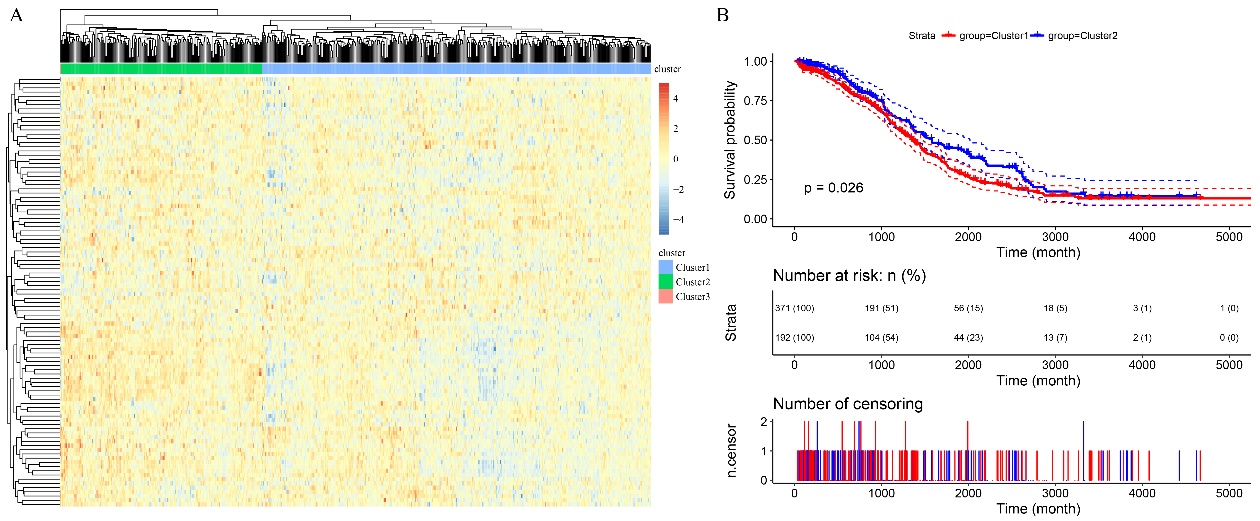

Supplement: Supplementary file 4 [file Table_4.docx]
