# Supplementary material for: Identification of a Prognostic Signature Associated With DNA Repair Genes in Ovarian Cancer
Source: Front Genet. 2019 Sep 12;10:839. doi: 10.3389/fgene.2019.00839 (PMC6751318; doi:10.3389/fgene.2019.00839)
Supplement: Supplementary file 5 [file Table_5.docx]

**Supplementary S5.** The top 20 DNA repair genes related to prognosis

| **Gene** | **p.value** | **HR** | **Low 95%CI** | **High 95%CI** |
| --- | --- | --- | --- | --- |
| FANCG | 0.000484 | 0.75668 | 0.64699 | 0.884968 |
| GTF2H4 | 0.00088 | 0.719155 | 0.592189 | 0.873343 |
| FANCI | 0.001165 | 0.816309 | 0.722192 | 0.922693 |
| SMUG1 | 0.002221 | 0.75754 | 0.634069 | 0.905054 |
| TDP1 | 0.002722 | 0.741544 | 0.609851 | 0.901676 |
| SSBP1 | 0.004855 | 0.69958 | 0.545583 | 0.897045 |
| PALB2 | 0.005858 | 0.72312 | 0.574211 | 0.910644 |
| POLD2 | 0.006912 | 0.76929 | 0.635967 | 0.930563 |
| BLM | 0.007262 | 0.818016 | 0.706432 | 0.947225 |
| FEN1 | 0.011141 | 0.800583 | 0.674248 | 0.950591 |
| DMC1 | 0.016831 | 0.456224 | 0.239731 | 0.868224 |
| JUNB | 0.01718 | 1.15757 | 1.026308 | 1.305621 |
| POLE | 0.018998 | 0.751938 | 0.592543 | 0.954211 |
| CHEK1 | 0.022643 | 0.854853 | 0.747012 | 0.978262 |
| FANCA | 0.024984 | 0.540501 | 0.315624 | 0.9256 |
| XRCC2 | 0.026376 | 0.820616 | 0.689221 | 0.977061 |
| MUTYH | 0.027438 | 0.816591 | 0.682018 | 0.977717 |
| RAD54L | 0.027754 | 0.806424 | 0.665808 | 0.976736 |
| MCM6 | 0.028526 | 0.852763 | 0.739472 | 0.983411 |
| RECQL | 0.032636 | 1.230636 | 1.017299 | 1.488712 |
