# Supplementary material for: Identification of a Prognostic Signature Associated With DNA Repair Genes in Ovarian Cancer
Source: Front Genet. 2019 Sep 12;10:839. doi: 10.3389/fgene.2019.00839 (PMC6751318; doi:10.3389/fgene.2019.00839)

**Supplementary S6.** The evaluation of prognostic signature based on external RNA-Seq data (TCGA, the July version). (a) ROC analysis of the prognostic signature. (b) The Kaplan-Meier survival analysis of the prognostic signature.


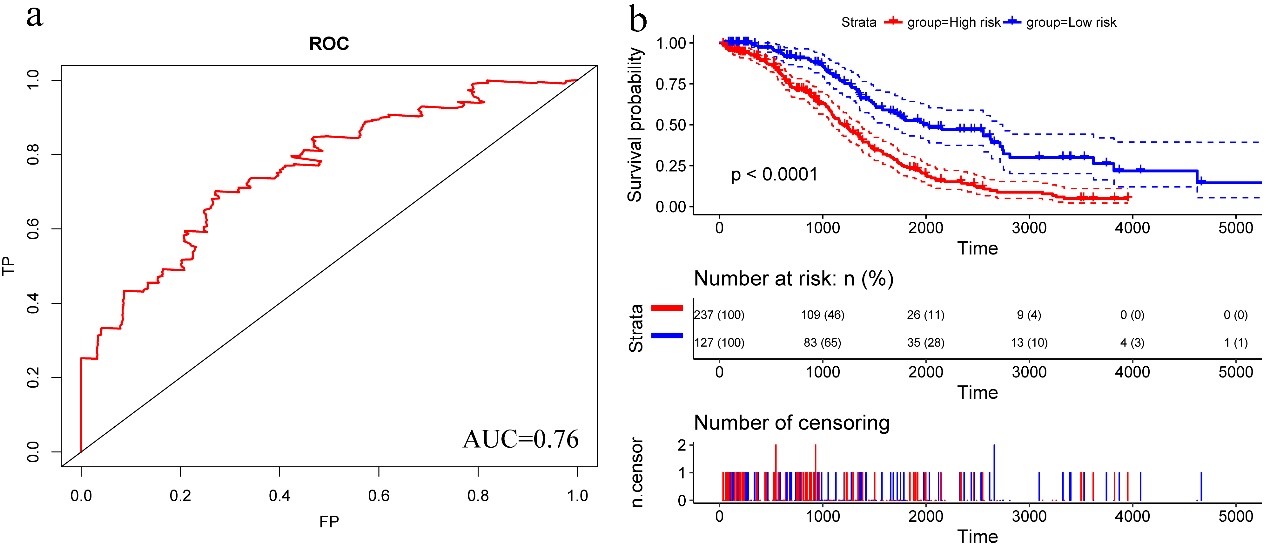

Supplement: Supplementary file 6 [file Table_6.docx]
