# Supplementary material for: Identification of a Prognostic Signature Associated With DNA Repair Genes in Ovarian Cancer
Source: Front Genet. 2019 Sep 12;10:839. doi: 10.3389/fgene.2019.00839 (PMC6751318; doi:10.3389/fgene.2019.00839)
Supplement: Supplementary file 7 [file Table_7.docx]

**Supplementary S7.** Characteristics of ovarian cancer patients

| **Characteristic** | **No.** | **%** |
| --- | --- | --- |
| **Age (y), median (range)** | 51(25-73) | 100% |
| **Stage** |  |  |
| I–II | 48 | 24.0% |
| III | 97 | 48.5% |
| IV | 55 | 27.5% |
| **Histological subtype** |  |  |
| Serous | 146 | 73.0% |
| Endometroid | 17 | 8.5% |
| Clear cell | 15 | 7.5% |
| Mullerian ductal | 22 | 11.0% |
| **Differentiation grade** |  |  |
| G1 | 45 | 22.5% |
| G2 | 53 | 26.5% |
| G3 | 68 | 34.0% |
| Unknown | 34 | 17.0% |
| **Residual disease** |  |  |
| optimal | 102 | 51.0% |
| suboptimal | 41 | 20.5% |
| unknown | 57 | 28.5% |
